# Occurrence and persistence of pseudo‐tail spots in the barn swallow

**DOI:** 10.1002/ece3.11669

**Published:** 2024-07-11

**Authors:** Masaru Hasegawa

**Affiliations:** ^1^ Department of Environmental Science Ishikawa Prefectural University Nonoichi Ishikawa Japan

**Keywords:** *Hirundo rustica gutturalis*, melanin pigmentation, ornamentation, sexual conflict, sexual selection

## Abstract

While numerous studies have confirmed sexual selection for ornamental traits in animals, it remains unclear about how animals exaggerate ornamentation across traits. I found that some Asian barn swallows *Hirundo rustica gutturalis* possessed “pseudo‐tail spots” on their undertail coverts adjacent to a well‐known sexual signal, the white tail spots. A close inspection showed their remarkable resemblance, and, as a consequence, pseudo‐tail spots appear to add white spots to the uniformly black central tail feathers, increasing the total number and area of white spots when spread tails are viewed from below. Pseudo‐tail spots on the undertail covers do not incur any flight cost, unlike the white tail spots on the tail itself, and thus presence of pseudo‐tail spots can represent an initial stage of a deceptive elaboration as predicted by sexual selection theory (i.e., males can elaborate traits with no additional flight cost, uncoupling flight cost and trait expression). The frequency of pseudo‐tail spots in the study population remained low even a decade after the first observation (ca. 7%), but was higher compared to other populations (e.g., 1% in another Japanese population). The slow progress of evolution, perhaps due to the low detectability of the trait, provides a unique opportunity to observe contemporary evolution of ornament exaggeration across traits. Further research with wider spatial and temporal coverage is needed to better understand the evolutionary and ecological importance of the trait.

## Graphical abstract

I found that some Asian barn swallows *Hirundo rustica gutturalis* possessed “pseudo‐tail spots” in their undertail coverts. A close inspection showed its remarkable resemblance to the white tail spots, a well‐known sexual signal in this species, although pseudo‐tail spots in the undertail covers do not incur any flight cost, unlike the white tail spots on the tail itself.

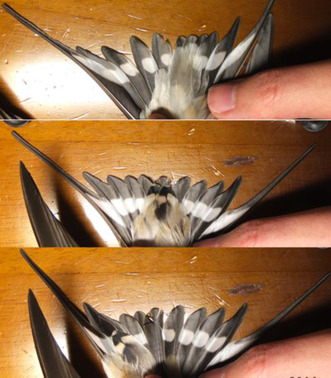

## 1 INTRODUCTION

Animals often exhibit ornamental traits that appear to have little viability function. Since Darwin first proposed the idea of sexual selection (Darwin, 1859, 1871), numerous empirical studies have revealed that animal ornamentation is, in fact, sexually selected through mate preference and intrasexual contest (reviewed in Andersson, 1994; Brooks & Griffith, 2010; Hill, 2006). One prevailing explanation for sexual selection posits that ornaments evolve because they convey information of signalers and thus signal receivers benefit from paying attention to them (i.e., honest signal; reviewed in Griffith & Pryke, 2006). Although empirical studies elucidate the evolution of specific ornamental traits (e.g., tail length; Møller, 1994; see also Svensson & Gosden, 2007 for contemporary evolution of these ornaments), many animals exhibit gaudy, complex ornamentation across multiple traits (e.g., peacock's long train adorned with multicolored eyespots: Dakin & Montgomerie, 2013). It remains elusive about the elaboration process across traits, that is, how animals exaggerate ornamentation toward extreme, elaborated forms (e.g., via honest signaling or other processes) after the occurrence of the original, rudimentary signals.

Theoretically, ornaments can be elaborated through sexual conflict (i.e., via diverging interests of males and females, here). This is because, whereas female interests are served by costly ornaments that reliably signal the quality of males (see above), male interests are to undermine the association between ornament expression and their quality to efficiently attract females (e.g., van Doorn & Weissing, 2006). In fact, about three decades ago, Hill (1994) had verbally theorized that once female mate preference for a costly signal spreads in a population, there will be strong selection on males to express the display character, sometimes novel traits, at “reduced cost” regardless of their actual quality (i.e., by uncoupling quality and trait expression, males can deceive females about the benefits she is receiving; see also van Doorn & Weissing, 2006 for mathematical models). This “deceptive” trait of signalers, or unreliable signal of quality compared to the ancestral state (sensu Hill, 1994; see also Bradbury & Vehrencamp, 1998), can rapidly spread through a population, which allows all individuals to converge on the maximum expression. However, the rapid fixation makes it challenging to observe the elaboration process within a single species, necessitating reliance on phylogenetic comparative analysis (Hill, 1994).

Here, I found the potential evolution of such deceptive elaboration in the Asian barn swallow, *Hirundo rustica gutturalis*. In barn swallows, the size of white tail spots is related to several indices of mate preference, including early breeding onset, high within‐pair paternity, and differential maternal investment, indicating intersexual selection for the ornament (Hasegawa et al., 2010, 2012; Kose et al., 1999; Kose & Møller, 1999; reviewed in Hasegawa, 2018). A recent meta‐analysis shows that white tail spots are an especially important sexual trait in the Asian subspecies (i.e., intense sexual selection there) compared to the European subspecies *H. r. rustica* (Romano et al., 2017). This is further corroborated by the finding that the Asian subspecies exhibit greater sexual dimorphism in the size of white tail spots compared to European subspecies (Hasegawa et al., 2017). At the same time, white tail spots exhibit inherent vulnerability to feather breakage because of structural weakness as they lack melanin pigments that strengthen feathers (Kose & Møller, 1999) and because of feather damage caused by feather lice, *Hirundoecus malleus*, which preferentially feeds on white feathers (Kose et al., 1999). Breakage of flight feathers (i.e., tail feathers, here) is more prevalent in individuals of lower quality (i.e., shorter‐tailed males with smaller white tail spots) and this damage reduces aerodynamic efficiency (Barbosa et al., 2002; Kose et al., 1999; Kose & Møller, 1999), indicating that large white tail spots are an honest signal of male quality, partially mediated by its influence on flight performance (Kose et al., 1999; Kose & Møller, 1999). Saino et al. (2015) demonstrated the condition‐dependence of the size of white tail spots (i.e., white spot size reflects body condition at molt), further supporting its role as a reliable quality indicator.

I report here that some barn swallows exhibit pseudo‐tail spots on the undertail coverts, which partially overlap the ventral surface of tail feathers and thus occur at similar position with white tail spots, and that the presence of pseudo‐tails spots has been persisted in the population for a decade since the first discovery. Notably, these spots are not located on flight feathers and, therefore, do not incur any flight cost (i.e., uncoupling flight cost and trait expression; see above). Potential adaptive and nonadaptive explanations for the evolution and maintenance of this novel trait and its implication in the elaboration process of ornamentation are discussed.

## 2 METHODS

**FIGURE 1:**
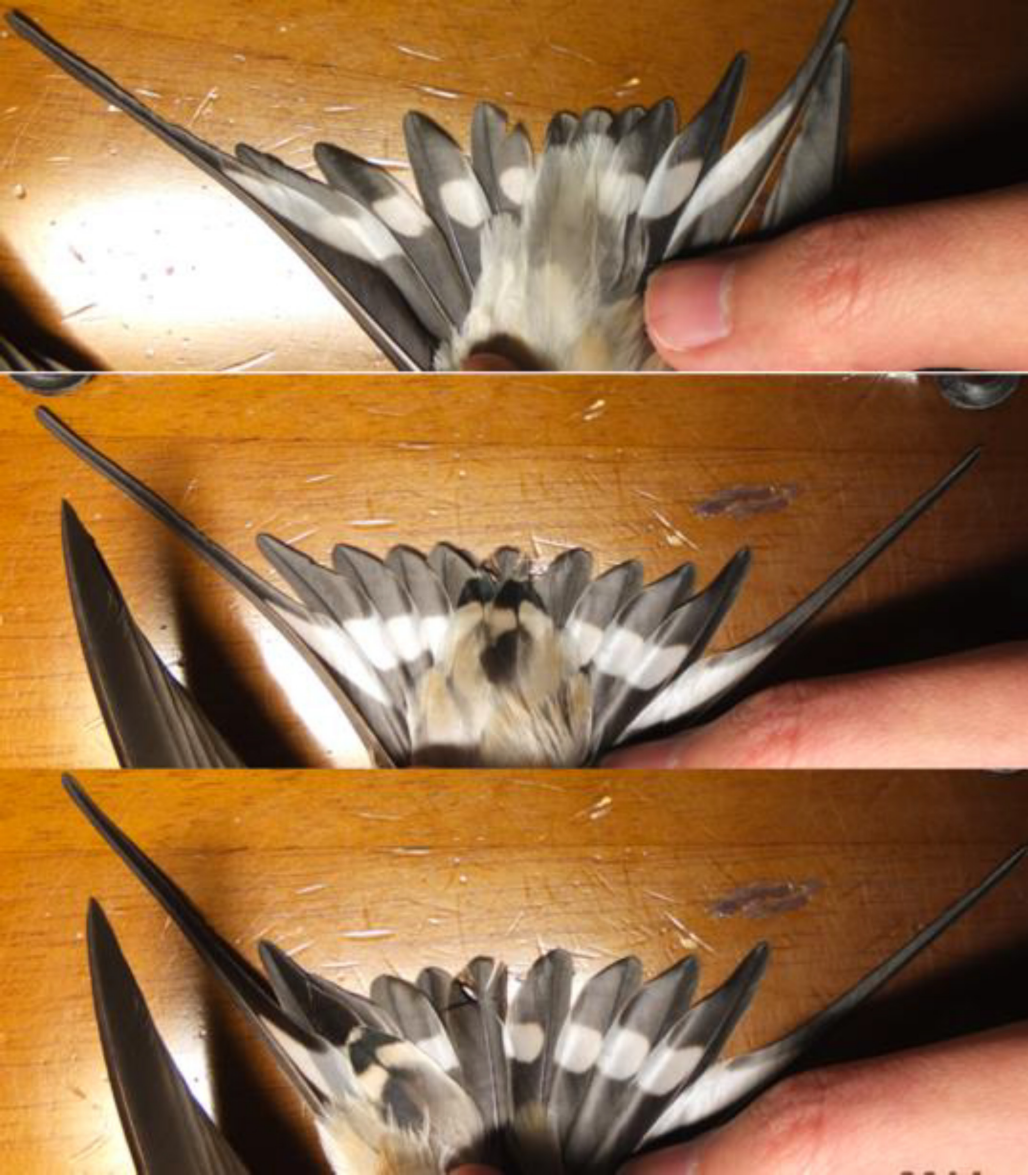
Pseudo‐tail spots found in a male Asian barn swallow, *Hirundo rustica gutturalis* in the Miyazaki prefecture in 2014 (upper panel: undertail coverts of typical male; middle panel: pseudo‐tail spots found on undertail coverts of the focal male; lower panel: pseudo‐tail spots were moved aside, revealing the central tail feathers that lack white spots).

During January 19–20, 2014, I captured and ringed a total of 28 post‐molting adult barn swallows (14 males and 14 females) at night, using sweep nets at their roost in Miyazaki City, Miyazaki Prefecture, Japan (31°54′ N, 131°25′ E). This population consists of barn swallows that are breeding in the same sites and thus can be regarded as resident (Arai et al. in prep; Hasegawa, 2020). The sex of swallows was determined from morphological measurements (e.g., males have longer tails; see Turner, 2006) and all adults were successfully sex‐identified. Among the 28 captured adults, two individuals exhibited pseudo‐tail spots on their undertail coverts, which are typically whitish in Asian barn swallows irrespective of age (adult or juvenile, here) or sexes (see Hasegawa, 2005 for detailed feather coloration in a breeding population at Niigata Prefecture). Unfortunately, I paid little attention in the first individual at the capture (resultantly, I failed to make a note to clarify which individual is this, which makes the sex of this individual unknown), and thus I focused on describing the second individual in greater detail (see Section 3: Results). In barn swallows, the two central tail feathers lack white spots (Figure 1, lower panel), which may be effective for flashing the white spots on their other tail feathers when spread (sensu JabŁoński, 1999), and thus I focused particularly on ventral view when they spread tails. To investigate the change in the frequency of pseudo‐tail spots 10 years later, I conducted a follow‐up survey in the same population during February 2023 and 2024 (specifically, February 12–17, 2023 and February 14–18, 2024), employing the same methodology used in the initial observation in 2014 (see above). This follow‐up survey aimed to test the prediction of rapid fixation of deceptive ornamentation, expecting an increase in the frequency of pseudo‐tail spots (see Section 1: Introduction). During 2024, two females out of 17 captured adult swallows exhibited pseudo‐tail spots, whereas none of the 15 adult swallows captured in 2023 exhibited pseudo‐tail spots.

Since the survey in Miyazaki Prefecture involved a wintering population (possibly including some migrants in addition to residents), I conducted an additional field survey during the breeding season (March–May) of 2014–2015 in a breeding population around Hayama‐cho, Kanagawa Prefecture, Japan (35°16′ N, 139°35′ E; see Hasegawa et al., 2017 for a detailed explanation of the study population). The sex of swallows was determined from morphological measurements. During 2014 and 2015, one female and one male, among the 110 and 103 captured adult swallows, respectively, exhibited pseudo‐tail spots on their undertail coverts.

## 3 RESULTS

Unlike typical males that have whitish undertail coverts, which obscure the white spots on the tail (Figure 1, upper panel), the focal male exhibited undertail coverts that closely resembled the white tail spots on the tail (i.e., “pseudo‐tail spots”; Figure 1, middle panel). Pseudo‐tail spots can conceal the black central tail feathers (i.e., those lacking white spots; Figure 1, lower panel), increasing the number (and thus total area) of white spots, when spread tails are viewed from below (see the difference between Figure 1, middle and lower panels). The proportion of individuals with pseudo‐tail spots in the captured birds in the Miyazaki population was 7.1% (Table 1; see Section 2: Methods). Close‐up pictures of the undertail covert feathers of the focal individual alongside 10 additional swallows (five males and five females) captured during the same season in the same population can be found in Figure S1, showing a notable difference between feathers with and without pseudo‐tail spots.

**TABLE 1:** Frequency of individuals with and without pseudo‐tail spots in the Miyazaki population of the Asian barn swallow.

	2014	2023 + 2024	Total
Males			
With pseudo‐tail spots	2 (or 1)	0	2 (or 1)
Without pseudo‐tail spots	12 (or 13)	14	26 (or 27)
Total	14	14	28
Females			
With pseudo‐tail spots	0 (or 1)	2	2 (or 3)
Without pseudo‐tail spots	14 (or 13)	14	28 (or 27)
Total	14	16	30
Males + females			
With pseudo‐tail spots	2	2	4
Without pseudo‐tail spots	26	28	54
Total	28	30	58

*Note*: Data from 2023 and 2024 were combined due to small sample sizes (2023: 0 out of 15 (*n*_males_ = 8; *n*_females_ = 7), 2024: 2 (*n*_males_ = 0; *n*_females_ = 2) out of 17 (*n*_males_ = 8; *n*_females_ = 9); note that two males were recaptured across years and thus total number of different individuals were 30). Figures in parentheses indicate females (note that one individual with pseudo‐tail spots in 2014 was failed to be identified in 2014; see Section 2: Methods).

**FIGURE 2:**
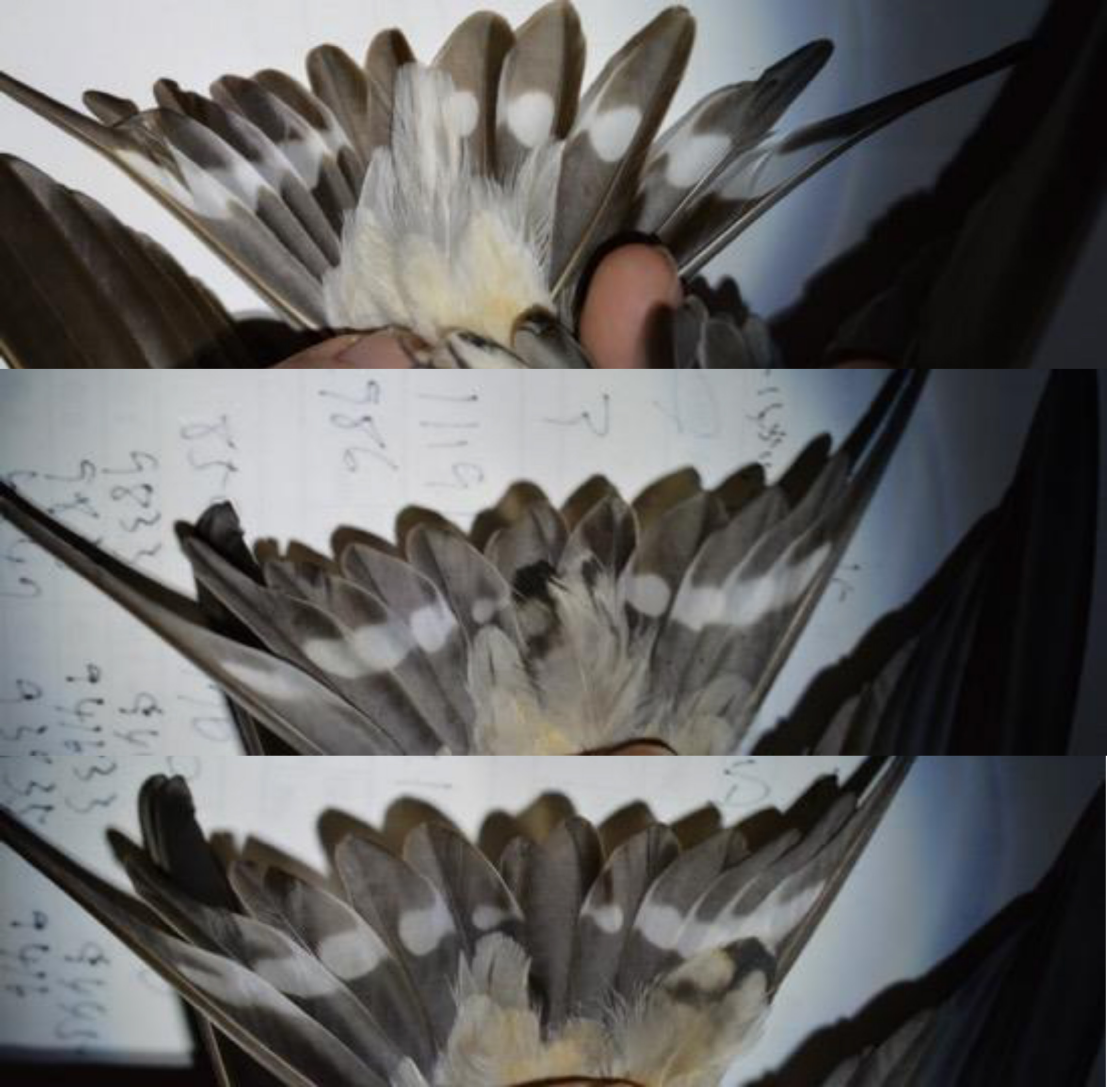
Pseudo‐tail spots found in a female Asian barn swallow, *Hirundo rustica gutturalis* in the Miyazaki Prefecture in 2024 (upper panel: undertail coverts of typical female; middle panel: pseudo‐tail spots found on undertail coverts of the focal female; lower panel: pseudo‐tail spots were moved aside, revealing the central tail feathers that lack white spots).

Ten years (2023–2024) after the initial 2014 observation of individuals with pseudo‐tail spots, I conducted a follow‐up survey in the same population. I found two females exhibiting pseudo‐tail spots in this period (Table 1, Figure 2; also see Figure S2 right panel for another female). The proportion of individuals with pseudo‐tail spots in this period was 6.7%, which was not significantly different from that observed in 2014 (Fisher's exact test, *p* = 1.00).

Pseudo‐tail spots were found in the breeding population of Hayama‐cho in Kanagawa prefecture, too. During a capture survey in 2014, one female (out of 110 individuals; 0.9%) exhibited pseudo‐tail spots (Figure S3). In 2015, one male (out of 103 individuals; 1.0%) exhibited the same feature (Figure S2 left panel). The frequency of individuals with pseudo‐tail spots in the breeding population did not differ significantly between 2014 and 2015 (Fisher's exact test, *p* = 1.00).

After pooling all individuals in each of the two populations, frequency of pseudo‐tail spots was significantly higher in the Miyazaki population (6.9%; Table 1) compared to the Hayama population (1.0%; male: 1/96; females: 1/94; Fisher's exact test, *p* = .028; note that we counted the number of different individuals to avoid pseudoreplication).

## 4 DISCUSSION

The main finding of the current study is the occurrence and persistence of pseudo‐tail spots on undertail coverts that closely resemble white tail spots, in the Miyazaki population of Asian barn swallows. Although black marking against white undertail coverts, such as shaft streaks, tips, or spots on undertail coverts have been reported as an abnormal form in barn swallows (e.g., see page 22 in Brombach, 2004 for detailed illustrations, which are replicated from Brombach, 1984, figures 2 and 3; also see Pedler, 1977; reviewed in Cramp, 1988; note that these are not white tail spots‐like shape), the current study presents the first record of pseudo‐tail spots, in which white spots distinctly appear against the black background of the undertail coverts. Because of the complex color patterning of the pseudo‐tail spots (see Figure S1 for the actual shape), simple abrasion resistance by melanic feather tips (reviewed in Bortolotti, 2006) is unlikely to explain the occurrence of pseudo‐tail spots. Possible explanations for the occurrence of pseudo‐tail spots include (1) genetic misexpression, that is, genes responsible for tail feather pigmentation pattern could become mistakenly expressed on the undertail coverts; (2) gene introgression from other species (e.g., interbreeding with other hirundine species, such the Pacific swallows *Hirundo tahitica*, which have black undertail coverts, might introduce these genes, though undertail coverts with white tail spots‐like feather patterning are absent at least in the genus *Hirundo*: Turner & Rose 1994); and (3) random mutation, that is, de novo mutations causing melanization on typically white undertail coverts. Although I could not distinguish these and possible other causations, environmental factor alone (e.g., stains and wears) cannot adequately explain the complex (and consistent) feather patterning (e.g., see Arai et al., 2015; Figuerola & Senar, 2005 for no detectable seasonal color change in eumelanin‐based feather coloration and no detectable seasonal eumelanin depigmentation, respectively), suggesting the genetic control of this novel trait, which is further reinforced by its persistence in the population a decade after its initial observation. Because white tail spots in Asian barn swallows serve as sexual signals at least in males (reviewed in Romano et al., 2017), the emergence of pseudo‐tail spots can be regarded as the initial stage of the elaboration process of ornamentation beyond the original trait (i.e., the “t3” stage in Figure 1 in Hill, 1994; note that this does not necessarily mean immediate viability or sexual functions of the novel trait; see below).

The occurrence and persistence of a new trait do not always imply it is adaptive. In addition to several adaptive explanations including sexual selection (see Section 1: Introduction), nonadaptive mechanisms, such as genetic drift and hitchhiking, could explain the persistence of neutral or even slightly maladaptive traits (Bergstrom & Dugatkin, 2016) and it remains unclear about whether barn swallows inherit pseudo‐tail spots across generations from the current study (i.e., its heritability remains untested). However, the population persistence for a decade, together with the observed population difference in the frequency (see Section 3: Results; note that wintering swallows in the Miyazaki population have particularly large white tail spots; Arai et al. in prep.), indicates that they would not be under a complete random process. This is further reinforced by the absence of similar observations in the nominate subspecies, despite extensive research on this well‐studied model system in the field of behavioral ecology (e.g., as we noted above, none of the black markings on undertail coverts illustrated in Brombach, 1984, which are representatives out of 326 examined individuals, resemble pseudo‐tail spots; reviewed in Møller, 1994; Turner, 2006; though this does not mean a complete absence of pseudo‐tail spots there). It is premature to conclude whether or not the observed pattern is maintained by selection on either sex (or both sexes), but it is likely that genetic mechanism that caused pseudo‐tail spots (or, in other words, failure to maintain typically whitish undertail coverts) could potentially become a target of selection.

Given that the fixation of additional ornamental traits is expected to be a rapid process (Hill, 1994), it is intriguing to see the low frequency of pseudo‐tail spots even after 10 years. This is particularly curious, considering that sexual selection on white tail spots are intense in the Asian subspecies (Romano et al., 2017), and that pseudo‐tail spots incur negligible flight cost, unlike tail feathers (Gill, 2007; see Section 1: Introduction). However, undertail coverts cannot be seen from above due to their positioning and thus would have low detectability (sensu Schluter & Price, 1993; see also Tazzyman et al., 2014). The low detectability likely hinders the effectiveness of pseudo‐tail spots as signals for signal receivers (and eavesdroppers), potentially explaining the lack of fixation of this trait.

In summary, some barn swallows exhibit pseudo‐tail spots on their undertail coverts, which closely resemble white tails spots. The occurrence and persistence of pseudo‐tail spots suggests the potential for exaggeration of existing sexual signals (i.e., white tail spots, here). Unlike theoretical prediction, the spread of pseudo‐tail spots appears slow, possibly due to their low detectability. This makes the white spots on both the tail and undertail coverts of barn swallows a rare opportunity to directly observe how costly sexual signal can exaggerate across traits in the wild. Wider spatiotemporal survey on the frequency of pseudo‐tail spots (e.g., full geographic range of species for several decades) and its relationship with selection pressure on the size of white tail spots remains to be conducted in the future.

## AUTHOR CONTRIBUTIONS

**Masaru Hasegawa:** Conceptualization (equal); formal analysis (equal); funding acquisition (equal); investigation (equal); validation (equal); visualization (equal); writing – original draft (equal); writing – review and editing (equal).

## Supporting information


Figure S1.‐S3.

## Data Availability

All data can be found in the main text.
