# Supplementary material for: Occurrence and persistence of pseudo‐tail spots in the barn swallow
Source: Ecol Evol. 2024 Jul 11;14(7):e11669. doi: 10.1002/ece3.11669 (PMC11239207; doi:10.1002/ece3.11669)

**Fig. S1** Single undertail coverts feathers plucked from the focal male (top left) and ten additional swallows from Miyazaki Prefecture, 2014. Left: feathers from males. Right: feathers from females.


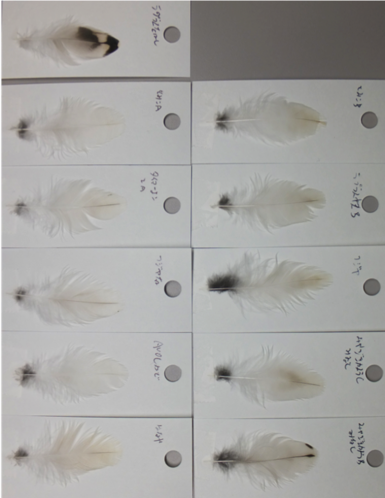


**Fig. S2** Additional examples of pseudo-tail spots in Asian barn swallows, *Hirundo rustica gutturalis*. Top left: male, Hayama population, 2015. Bottom left: the same individual as upper left. Right: female, Miyazaki population, 2024. Note that central tail feathers lack white spots.


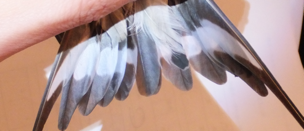

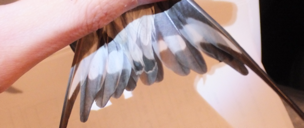

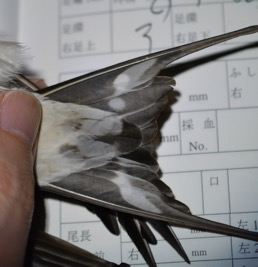


**Fig. S3** Single undertail covert feather from a female barn swallow in the Hayama population, Kanagawa Prefecture, 2014.


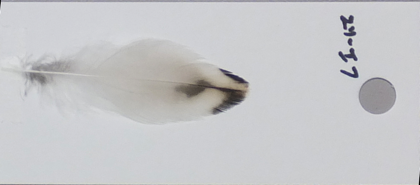

Supplement: Supplementary file 1 — Figure S1.‐S3. [file ECE3-14-e11669-s001.docx]
